# Explore the Diagnostic Efficiency of Chinese Thyroid Imaging Reporting and Data Systems by Comparing With the Other Four Systems (ACR TI-RADS, Kwak-TIRADS, KSThR-TIRADS, and EU-TIRADS): A Single-Center Study

**DOI:** 10.3389/fendo.2021.763897

**Published:** 2021-10-27

**Authors:** Qi Qi, Aiyun Zhou, Suping Guo, Xingzhi Huang, Songli Chen, Yaohui Li, Pan Xu

**Affiliations:** Department of Ultrasonography, The First Affiliated Hospital of Nanchang University, Nanchang, China

**Keywords:** thyroid nodule, diagnostic imaging, ultrasonography, risk assessment, C-TIRADS

## Abstract

**Purpose:**

To explore the characteristics of C-TIRADS by comparing it with ACR-TIRADS, Kwak-TIRADS, KSThR-TIRADS and EU-TIRADS.

**Methods:**

A total of 1096 nodules were collected from 884 patients undergoing thyroidectomy in our center between May 2018 and December 2020. Divided the nodules into two groups: “>10mm” and “≤10mm”. Ultrasound characteristics of each nodule were observed and recorded by 2 doctors, then classified based on ACR-TIRADS, Kwak-TIRADS, KSThR-TIRADS, EU-TIRADS, and C-TIRADS.

**Results:**

A total of 682 benign nodules cases (62.23%) and 414 malignant nodules cases (37.77%) were identified. The ICC value of each guideline was:0.937(ACR-TIRADS), 0.858(EU-IRADS), 0.811(Kwak-TIRADS), 0.835(KTA/KSThR-TIRADS) and 0.854(C-TIRADS). The nodule malignancy rates in the groups(Kwak-TIRADS 4B, C-TIRADS 4B、4C) of two sizes were significantly different (all p<0.05). There was no statistical difference in the other grades of two sizes (all p>0.05). Unnecessary biopsy rates were the lowest in C-TIRADS (49.02% p<0.001). Furthermore, Kwak-TIRADS had the highest sensitivity and NPV (89.9%, 91.0%, all p<0.05), while C-TIRADS had the highest specificity and PPV (82.3%, 69.2%, all p<0.05). C-TIRADS and Kwak-TIRADS had the highest accuracy (76.0%, 72.5%, P=0.071). The AUCs of the 5 guidelines were C-TIRADS(0.816, P<0.05), Kwak-TIRADS(0.789, P<0.05) KTA/KSThR-TIRADS and ACR-TIRADS(0.773, 0.763, P=0.305), EU-TIRADS(0.734, P<0.05). The AUCs of the five guidelines were not statistically different between “nodules>10mm” and “nodules ≤ 10mm” (all P>0.05).

**Conclusions:**

All five guides showed excellent interobserver agreement. C-TIRADS was slightly efficient than Kwak-IRADS, KTA/KSThR-TIRADS and ACR-TIRADS, and had greater advantages than EU-TIRADS. The diagnostic abilities of the five guidelines for “nodules ≤ 10mm” were not inferior to that of “nodules> 10mm”. C-TIRADS is simple and easy to implement and can provide effective thyroid tumor risk stratification for thyroid nodule diagnosis, especially in China.

## Introduction

Thyroid nodule is the most common thyroid gland disease. Thyroid and malignant nodules can be detected in over 50% and 7-15% of the general population, respectively (1). Ultrasound is the most commonly used and effective imaging method for thyroid nodule diagnosis. Ultrasound can show the morphological characteristics of thyroid nodules clearly, including nodules ≤10mm. The number of thyroid nodules and malignant thyroid nodules detected has been increasing yearly due to the increased use of thyroid ultrasonography. Presently, various guidelines are used to differentiate benign and malignant thyroid nodules in clinical practice and science. Previous research showed that each guideline has advantages and limitations (2–8).

The following guidelines are widely used in China : Kwak-TIRADS (Thyroid Imaging Reporting and Data System) developed by Kwak and published in Radiology in 2011 (9),KTA/KSThR-TIRADS developed by the Korean Society of Radiology and the Korean Society of Thyroid Radiology In 2016 (10), ACR-TIRADS guideline, developed by the American College of Radiology in 2017 (ACR) (11), and EU-TIRADS created by European Thyroid Association (2017) (ETA) (12). The above guidelines are used to detect malignant probability based on the ultrasound characteristics of thyroid nodules to guide further treatment. These guidelines indicate similar suspicious features of thyroid nodules, such as solid, hypoechoic, marked hypoechoic, irregular margin, and microcalcification. However, the specific ultrasound characteristics, counting, and grading methods are different FNA(fine needle aspiration) recommendations are also different (12).

The use of TIRADS guidelines in China is not yet uniform and can cause doubts to clinicians and patients (13). Furthermore, ultrasound doctors face some challenges during diagnosis. For instance, ACR-TIRADS assigns scores to about 18 ultrasound features, which is ineffective for diagnosis and can reduce the efficiency of diagnosis in a country with a large population. The malignancy rates of the highest grades of ACR-TIRADS and EU-TIRADS are >20% and 26%-87%, respectively, the malignant rates range corresponding to the highest grades were too large, which confusing clinicians on the treatment of thyroid nodules. One more malignant feature can make the classification reach 4C and 5 when using Kwak-TIRADS and EU-TIRADS guidelines for solid hypoechoic nodules. These TIRADS guidelines also guide FNA. However, it is not realistic to conduct FNA before determining every treatment plan in China since it is not widely developed in China.

The Superficial Organ and Vascular Ultrasound Group in the Chinese Medical Association issued a new guideline, C-TIRADS(Chinese Thyroid Imaging Reporting and Data Systems), in August 2020 to solve the above problems (13). C-TIRADS is a new counting classification method used for thyroid nodule diagnosis and guiding thyroid FNA. It takes into account both the international standards and China’s national conditions. Presently, few studies have reported on C-TIRADS. This study aimed to explore the characteristics of C-TIRADS by comparing it with ACR-TIRADS, Kwak-TIRADS, KSThR-TIRADS and EU-TIRADS.

## Materials and Methods

This is a retrospective study. Informed consent was not required for this retrospective observational study.

### Patient Selection

**Figure 1:**
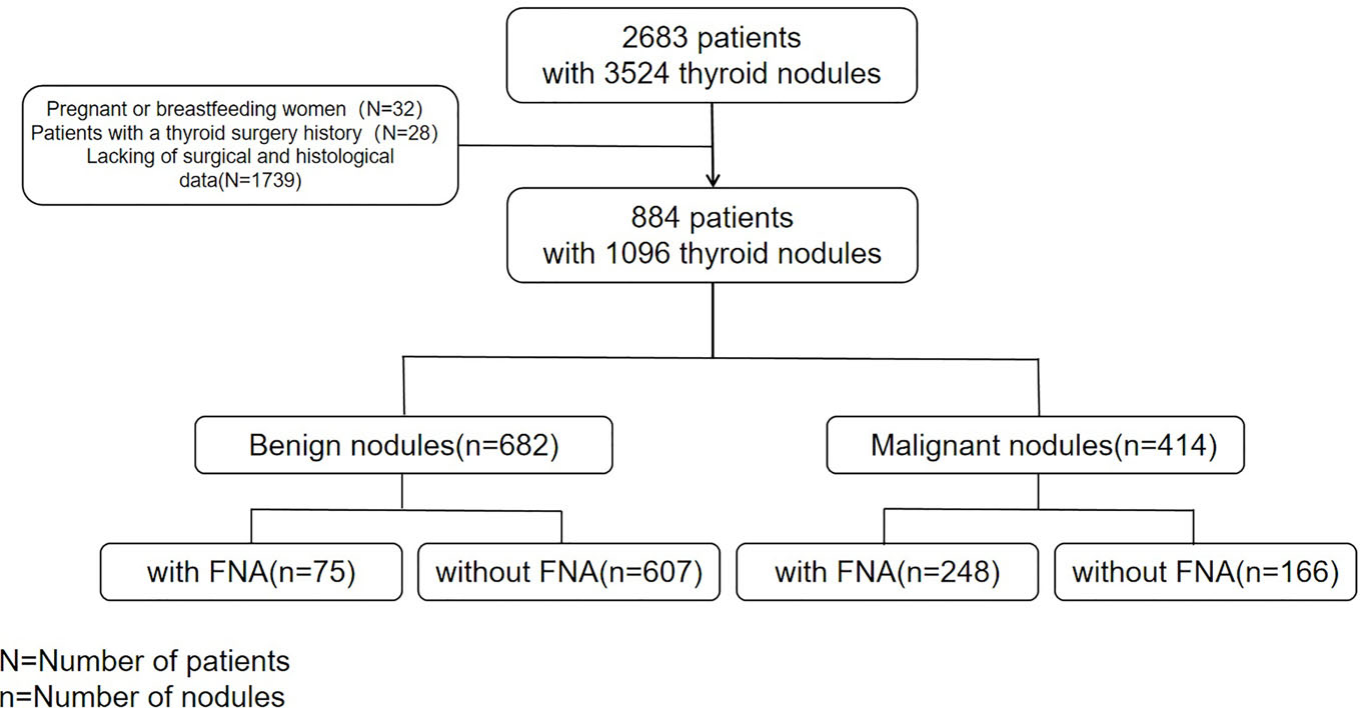
Flowchart of study participants.

From May 2018 to December 2020,two radiologists with over 5 and 7 years of experience in thyroid ultrasound diagnosis collected ultrasound images of 2683 consecutive patients with 3524 thyroid nodules in our hospital. The patients were followed up, except for pregnant and breastfeeding women and patients with a thyroid surgery history. Finally, 884 patients (1096 nodules) who underwent thyroidectomy (complete, almost complete, or unilateral thyroidectomy), had complete clinical data such as gender and age, ultrasound features data and surgical pathological results of thyroid nodules were included in the study (**Figure 1**).

### Thyroid Ultrasound Examination

All ultrasound examinations were performed with Phillip iu22, epiq7 or Toshiba aplio500 devices equipped with either a 5–12 MHz or a 10 MHz linear-array transducer. Two US experts who had 5 and 7 years of experience in performing thyroid US examination explored the thyroid region of the patients, stored the complete and clear thyroid nodules ultrasound images as JPEG files, recorded the location of the nodules (right/left lobe, isthmus), The three diameters of the nodes were measured three times and the average value was recorded. The three diameters of the nodes(upper and lower diameter, left and right diameter,front and back diameter) were measured three times and the average values were recorded. Recorded the maximum diameter of the nodules. Suspicious cervical lymph node metastasis was also observed.

### Nodule Analysis

The nodules without histological results were excluded after follow-up. Two US experts who had 10 and 15 years of experience in performing thyroid US examination and did not know the final histology of nodules retrospectively reviewed the images and independently analyzed all nodules. Only the nodules with clear pathological diagnoses were included when a patient had more than one nodule. Nodule composition (cystic, almost completely cystic, spongiform, mixed cystic and solid, solid or almost completely solid), echogenicity (no echo, hyperechoic, isoecho, hypoechoic, markedly hypoechoic), shape (wider-than-tall, taller-than-wide), Margin (well circumscribed, microlobulated or irregular, ill-defined, extra thyroid extension), and hyperechoic (microcalcifications, peripheral calcification, macrocalcifications, comet-tail sign) were recorded. The nodules were then classified based on Kwak-TIRADS (9), KSThR-TIRADS (10), ACR-TIRADS (11),EU-TIRADS (12), and C-TIRADS (2) guidelines. The results were compared, and the two doctors discussed and settled on a final result whenever there was a disagreement.

### Statistics

IBM SPSS Statistics (version 22) and R-Project (version 4.0.5) were used for statistical analyses. Quantitative data were presented as mean ± standard deviation (SD), while qualitative data were presented as frequencies. The Shapiro–Wilk test was used to determine the presence of a normal distribution. Differences between groups were analyzed using a Mann–Whitney U test for nonparametric data and an unpaired t-test for parametric data. The χ2 test or Fisher’s exact test was used to compare categorical variables. ICC (intraclass correlation coefficient)was used to evaluate inter-observer agreement. Unnecessary biopsy rates were calculated as the proportion of benign nodules among thyroid nodules that were indicated for biopsy in the five guidelines. The sensitivity, specificity, positive predictive value (PPV), negative predictive value (NPV), and accuracy were determined by comparing them with the pathological findings. Kendall’s tau-b test was used to assess the relationship between each category and the pathology findings. The receiver operating characteristic (ROC) curves of the four guidelines were used to calculate the best cut-off value. The DeLong test was used to compare the ROC curves *via* the pROC software package (“R-Project, version 4.0.5”). P < 0.05 was considered statistically significant.

## Results

### Characteristics of Patients and Nodules

A total of 884 patients (681 females and 203 males; median age, 43.91 years; between 10 and 78 years) were included in the study. A total of 507 benign cases (397 females and 110 males; median age, 49.26 years; between 25and 78 years)and 377 malignant cases (284 females and 93 males; median age, 41.83 years; between 10 and 73 years) were detected. Patients with thyroid cancer were significantly younger than those with benign nodules(P<0.001). The gender difference was not significant (P=0.299).

A total of 1096 thyroid nodules (average maximum diameter, 18.86 mm, between 5 and 64mm) were identified. There were 682 benign nodules (average maximum diameter, 19.13mm between 5 and 64mm)and 414 malignant nodules (average maximum diameter, 17.76mm between 5 and 60mm). The malignant nodules were smaller than the benign nodules (P=0.043).The pathological results of the nodules are shown in **Table 1**.

**Table 1:** Pathological results of 1096 thyroid nodules.

Pathology	n (%)
Benign	682 (62.23%)
Nodular goiter	473 (69.35%)
Normal follicular cells	17 (2.49%)
Follicular adenoma	148 (20.53%)
Inflammatory changes of thyroid gland	33 (4.84%)
Other benign lesions	11 (1.61%)
malignant	414 (37.77%)
Papillary carcinoma	384 (92.75%)
follicular carcinoma	10 (2.42%)
Medullary carcinoma	7 (1.69%)
Undifferentiated carcinoma	6 (1.45%)
Other malignant lesions	7 (1.69%)

FNA was performed on 332 nodules (248 malignant nodes and 75 benign nodes). 7 nodules were classified as BSRTC I, 58 as BSRTC II, 12 as BSRTC III, 12 as BSRTC IV, 104 as BSRTC V, and 139 as BSRTC VI.

### The Relationship Between the Classification of the Five Guidelines and Nodule Pathology

The incidence of malignancies of different grades in the five guidelines is shown in **Table 2**. The calculated malignancy rates of the following levels were higher than the recommended malignancy risks: ACR-TIRADS TR3 11.48% (2%-5%), ACR-TIRADS TR4 29.24% (5%-20%), EU-TIRADS4 22.86% (6%-17%), C-TIRADS4A 16.77% (2%-10%), and the calculated malignancy rates of the other levels were within the recommended malignancy risk range. The correlation coefficients of five guidelines were:0.442(ACR-TIRADS), 0.502(Kwak-TIRADS), 0.427(EU-TIRADS), 0.502(C-TIRADS), 0.467(KTA/KSThR-TIRADS).

**Table 2:** Malignancy rates in ACR-TIRADS,Kwak-TIRADS,EU-TIRADS, C-TIRADS, KTA/KSThR-TIRADS.

Classification	Total							TN≥10mm			TN<10mm			
	B (n)	M (n)	Total (n)	CMR (%)	RMR (%)	CC	P value	B (n)	M (n)	CMR (%)	B (n)	M (n)	CMR (%)	P value
ACR-TIRADS							<0.001							
TR1	40	0	40	0	<2	0.442		28	0	0	12	0	0	1.000
TR2	145	1	146	0.68	<2			125	1	0.79	20	0	0	1.000
TR3	54	7	61	11.48	2-5			31	5	13.89	23	2	8.00	0.763
TR4	242	100	342	29.24	5-20			202	81	28.62	40	19	32.20	0.582
TR5	201	306	507	60.36	>20			157	256	61.99	44	50	53.19	0.116
Kwak-TIRADS							<0.001							
2	40	0	40	0	0	0.502		28	0	0	12	0	0	1.000
3	149	1	150	0.67	≤5			128	1	0.78	21	0	0	1.000
4A	98	8	106	7.55	5-10			69	6	8.00	29	2	6.45	1.000
4B	136	33	169	19.53	10-50			110	21	16.03	26	12	31.58	0.033
4C	246	326	572	56.99	50-85			195	278	58.77	51	48	48.48	0.600
5	13	46	59	77.97	85-100			13	37	74.00	0	9	100	0.185
EU-TIRADS							<0.001							
2	40	0	40	0	0	0.427		28	0	0	12	0	0	1.000
3	203	9	212	4.25	2-4			161	7	4.19	42	2	4.55	1.000
4	135	40	175	22.86	6-17			105	26	19.85	30	14	31.82	0.102
5	304	365	669	54.56	26-87			249	310	55.46	55	55	50.00	0.293
C-TIRADS							<0.001							
2	44	0	44	0	0	0.502		36	0	0	8	0	0	1.000
3	187	2	189	1.06	<2			153	2	1.29	34	0	0	1.000
4A	139	28	167	16.77	2-10			100	16	13.79	39	12	23.53	0.121
4B	191	112	303	36.96	10-50			141	95	40.25	50	17	25.37	0.026
4C	121	263	384	68.49	50-90			113	224	66.47	8	39	82.98	0.022
5	0	9	9	100	>90			0	6	100	0	3	100	1.000
KTA/KSThR-TIRADS							<0.001							
2	74	0	74	0	<3	0.467		56	0	0	18	0	0	1.000
3	210	10	220	4.55	3-15			166	8	4.60	44	2	4.35	1.000
4	171	66	237	27.85	15-50			142	50	26.04	29	16	35.56	0.200
5	227	338	565	59.82	>60			179	285	61.42	48	53	52.48	0.096

B, benign nodules; M, malignant nodules; TN, thyroid nodule; CMR, calculated malignancy rate; RMR, recommended malignancy risk; CC, correlation coefficient.

Each guideline was divided into groups according to whether the nodule was greater or smaller than 10mm. The number of nodules and the incidence of malignant tumors in each group were calculated (**Table 2**). The nodule malignancy rates in the grades(Kwak-TIRADS 4B,C-TIRADS 4B,C-TIRADS 4C) of two sizes were significantly different (all p<0.05). And the malignancy rates of Kwak-TIRADS 4B were higher in “nodules ≤ 10mm” than in “nodules >10mm”. The malignancy rates of C-TIRADS 4B and C-TIRADS 4C were lower in “nodules ≤ 10mm” than in “nodules >10mm”. There was no statistical difference in the malignant rate of nodules in the other grades between “nodules ≤ 10mm” and “ nodules >10mm” (all P>0.05).

### Inter-Observer Agreement

**Figure 2:**
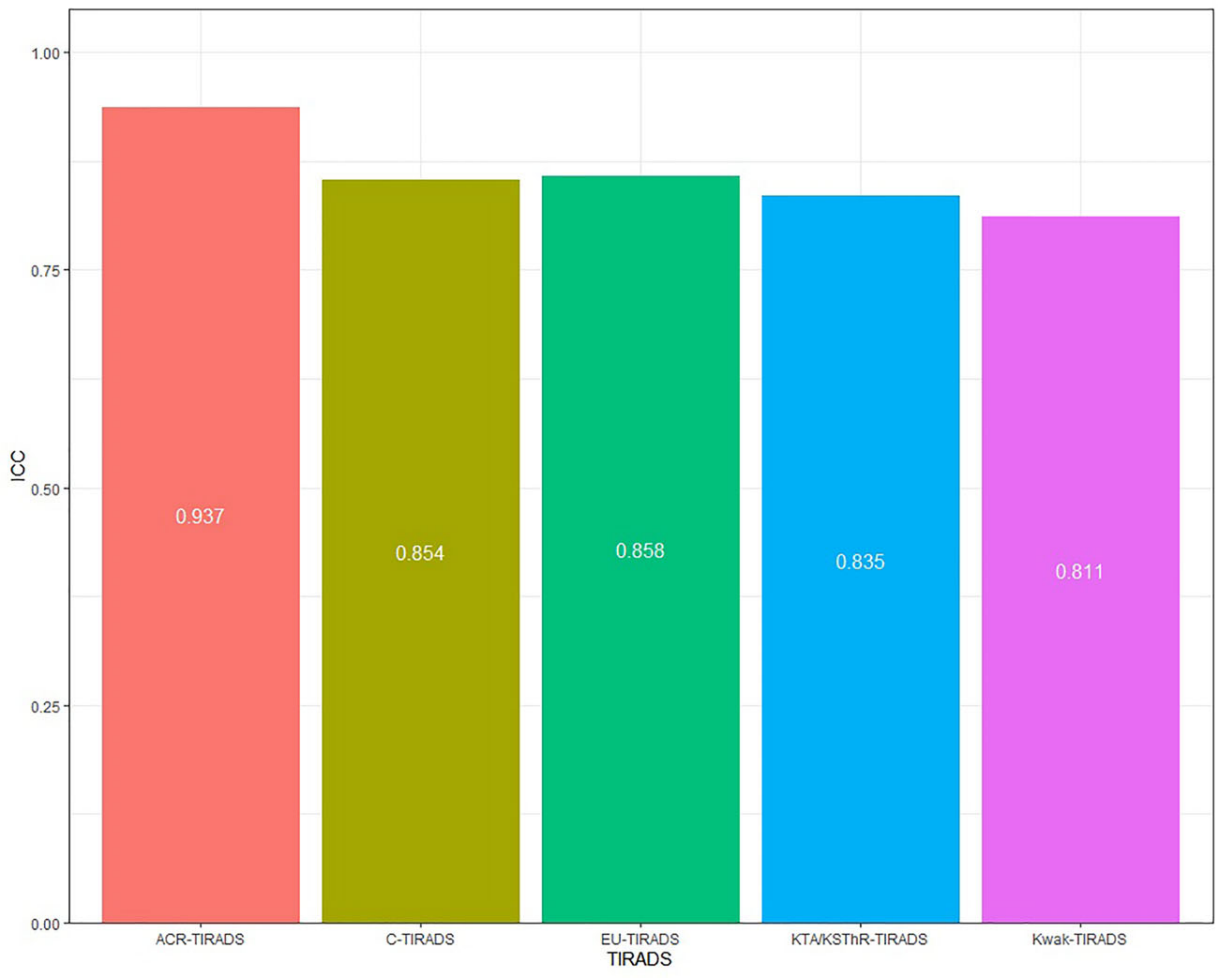
The ICC values of five guidelines.

**Figure 3:**
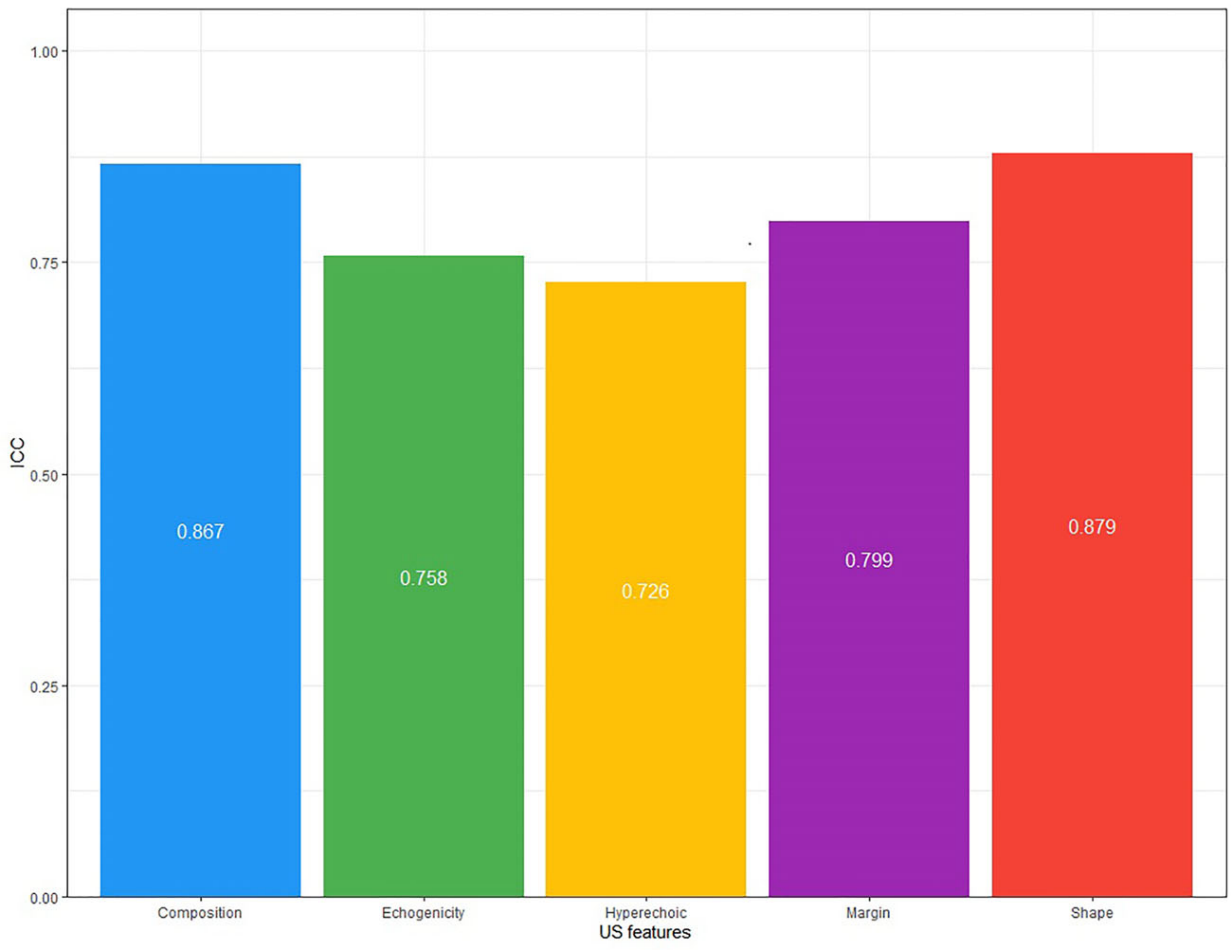
The ICC values of Ultrasound features.

Consistency analysis was conducted for the original data of the two doctors. The ICC value of each guideline was: 0.937(ACR-TIRADS), 0.858(EU-IRADS), 0.811(Kwak-TIRADS), 0.835(KTA/KSThR-TIRADS) and 0.854(C-TIRADS), with good repeatability (**Figure 2**). The ICC value of each US feature was: 0.867(composition), 0.758(echogenicity), 0.726(hyperechoic), 0.799(margin), 0.879(shape), with good repeatability (**Figure 3**).

### Unnecessary Biopsy Rate

**Supplementary Material 2** shows statistics on the FNA recommended by each guideline. If FNA was performed on each nodule that met the FNA indication in this study, their unnecessary biopsy rates are: ACR-TIRADS 50.25%, EU-TIRADS 55.99%, Kwak-TIRADS 53.09%,C-TIRADS 49.02%,KTA/KSThR-TIRADS 58.36%, (all p<0.001).

### Diagnostic Performance of the Five Guidelines

The diagnostic efficacy and the ROC curves of the five guidelines are shown in **Table 3**, respectively. ROC analysis showed that the best diagnostic cut-off values of ACR-TIRADS, Kwak-TIRADS, EU-TIRADS, KTA/KSThR-TIRADS and C-TIRADS were TR5, 4C, 5, 5and 4C, respectively. Kwak-TIRADS had the highest sensitivity and NPV (89.9%,91.0%, all P<0.05). C-TIRADS had the highest specificity, PPV (82.3%,69.2%, all P<0.05). C-TIRADS and Kwak-TIRADS had the highest accuracy (76.0%,72.5%,P=0.071). Furthermore, C-TIRADS had the highest AUC (0.816, all P<0.001), followed by Kwak-TIRADS (0.789, all P<0.05). There was no statistical difference between KTA/KSThR-TIRADS and ACR-TIRADS (0.773, 0.763, P=0.305). The AUC of EU-TIRADS was 0.734 (all P<0.001).

**Table 3:** Diagnostic performance of 5 guidelines.

Method	Cut-off	Sensitivity (%)	Specificity (%)	PPV (%)	NPV (%)	Accuracy (%)	AUC
ACR-TIRADS							
	5	73.9 (69.4-78.1)	70.5 (67.0-73.9)	60.4 (57.2- 63.4)	81.7 (79.09-84.1)	71.8 (69.0- 74.5)	0.763 (0.736-0.791)
P value		0.012^a^	<0.001^a^	0.007^a^	0.439^a^	0.029^a^	<0.001^a^;0.004^b^;0.305^c^
Kwak-TIRADS	4C	89.9 (86.5- 92.6)	62.0 (58.3- 65.7)	58.9 (56.5- 61.3)	91.0 (88.3- 93.1)	72.5 (69.8-75.2)	0.789 (0.763-0.815)
P value		<0.001^a^	<0.001^a^	0.001^a^	<0.001^a^	0.071^a^	<0.001^a^
EU-TIRADS	5	88.2 (84.7- 91.1)	55.4 (51.6- 59.2)	54.6 (52.3-56.8)	88.5 (85.5- 91.0)	67.8 (64.9- 70.6)	0.734 (0.705-0.763)
P value		<0.001^a^	<0.001^a^	<0.001^a^	<0.001^a^	<0.001^a^	<0.001^a^;<0.001^b^;<0.001^c^;<0.001^d^
KTA/KSThR-TIRADS	5	81.6 (77.6- 85.3)	66.7 (63.0-70.3)	59.8 (57.0- 62.6)	85.7 (82.9- 88.1)	72.4 (69.6- 75.0)	0.773 (0.746-0.800)
P value		<0.001^a^	<0.001^a^	0.037^a^	0.708^a^	0.043^a^	<0.001^a^;0.014^b^
C-TIRADS	4C	75.7 (70.9- 80.3)	82.3 (79.2- 85.1)	69.2 (65.3-72.8)	79.8 (77.45-81.9)	76.0 (73.4-78.5)	0.816 (0.791-0.841)

95% confidence intervals are in parentheses; PPV, positive predictive value; NPV, negative predictive value; AUC, area under the receiving operator characteristics curve; P value, ^a^Compared with C-TIRADS; ^b^Compared with Kwak-TIRADS; ^c^Compared with KTA/KSThR-TIRADS; ^d^Compared with ACR-TIRADS.

The diagnostic efficacy of the five guidelines for nodules with different sizes is shown in **Table 4**. The AUCs of the five guidelines were not statistically different between “nodules ≤ 10mm” and “nodules >10mm” (all P>0.05).

**Table 4:** Diagnostic performance of different sizes of 5 guidelines.

Method		Cut-off	Sensitivity (%)	Specificity (%)	PPV (%)	NPV (%)	Accuracy (%)	AUC
ACR-TIRADS	≤10mm	5	70.4 (58.4-80.7)	68.4 (59.9-75.9)	53.2 (46.0-60.2)	81.9 (75.6-86.8)	69.1 (62.3-75.2)	0.746 (0.681-0.811)
	>10mm	5	74.6 (69.7-79.2)	70.1 (66.0-74.0)	62.0 (58.5-65.3)	80.9 (77.8-83.7)	71.9 (68.8-74.9)	0.767 (0.737-0.798)
P value			0.025	0.235	0.537	0.052	0.859	0.513
Kwak-TIRADS	≤10mm	4C	80.3 (69.1-88.8)	63.3 (54.7-71.3)	52.8 (46.6-58.9)	86.3 (79.5-91.1)	69.1 (62.3-75.2)	0.780 (0.719-0.840)
	>10mm	4C	91.8 (88.4-94.5)	61.7 (57.5-65.8)	60.2 (57.5-62.9)	92.3 (89.3-94.5)	73.4 (70.3-76.3)	0.790 (0.761-0.819)
P value			0.007	0.801	0.185	0.094	0.240	0.721
EU-TIRADS	≤10mm	5	77.5 (66.0-86.5)	60.4 (51.78- 68.6)	50.0 (44.0-56.0)	84.0 (77.0-89.1)	66.2 (59.4-72.6)	0.726 (0.659-0.793)
	>10mm	5	90.3 (86.8-93.3)	54.1 (49.9-58.3)	55.5 (53.0-57.9)	89.9 (86.5-92.6)	68.2 (65.0-71.2)	0.734 (0.702-0.767)
P value			0.004	0.216	0.344	0.149	0.638	0.794
C-TIRADS	≤10mm	4C	69.2 (56.8-80.7)	94.2 (89.0-97.5)	84.0 (72.3-91.4)	81.9 (77.3-85.7)	82.4 (76.5-87.3)	0.833 (0.776-0.890)
	>10mm	4C	77.1 (71.8-82.0)	79.2 (75.5-82.5)	67.1 (63.0-70.9)	79.2 (76.5-81.7)	74.5 (71.5-77.3)	0.808 (0.780-0.836)
P value			0.254	<0.001	0.023	0.527	0.021	0.411
KTA/KSThR-TIRADS	≤10mm	5	74.7 (62.9-84.2)	65.5 (56.9-73.3)	52.5 (45.8-59.0)	83.5 (76.9-88.5)	68.6 (61.8-74.8)	0.750 (0.685-0.814)
	>10mm	5	83.1 (78.7-86.9)	67.0 (62.9-71.0)	61.4 (58.3-64.4)	86.3 (83.1-88.9)	73.3 (70.2-76.1)	0.779 (0.749-0.809)
P value			0.132	0.803	0.121	0.560	0.201	0.365

95% confidence intervals are in parentheses; PPV, positive predictive value; NPV, negative predictive value; AUC, area under the receiving operator characteristics curve; P value, Compared with C-TIRADS.

## Discussion

Most guidelines currently provide guidance for FNA, but FNA is not widely available in China, and it is not realistic to mandate FNA for every thyroid nodule before deciding on a treatment plan. Therefore, C-TIRADS guideline points out that in medical institutions that have not yet carried out FNA, the results of C-TIRADS may provide some suggestions for surgeons’ treatment decisions (13). Furthermore, Kwak-TIRADS and C-TIRADS were very similar, and both had the simplest counting method. However, C-TIRADS was different from other risk stratifications since it removed the “hypoechoic” and “mainly solid” characteristics from the malignant signs, indicating the “comet tail sign” as a benign sign and calculating it as -1 point. The Kwak-TIRADS and C-TIRADS grades of the same nodule were then compared. A total of 462 nodules were degraded due to echo and composition. For the number of high-grade nodules, a total of 393 high-grade nodules of C-TIRADS (C-TIRADS 4C+C-TIRADS 5) were identified, which was lower than EU-TIRADS 5 (669cases), Kwak-TIRADS (631 cases) (Kwak-TIRADS4C+Kwak-TIRADS 5), KTA/KSThR-TIRADS 5(565cases) and ACR-TIRADS TR5 (507 cases). The unnecessary puncture rate of C-TIRADS was lowest (49.02%,all p<0.001). Therefore, C-TIRADS can reduce the grade of nodules without affecting the puncture standards.

The benign and malignant nodules had different sizes, and the maximum diameter of benign nodules was larger than that of malignant nodules, consistent with Gao’s study (14). The difference could be due to selection bias since most benign patients undergo surgery due to oppressive symptoms and aesthetic needs. The malignancy rate of nodules in each guideline increased with the increase in the grade, indicating a correlation with guidelines. The malignancy rates of most grades in the 5 guidelines were within the range of malignancy rates recommended by each guideline, which shows that the sample in this study was representative. ACR-TIRADS TR3 (11.48%),ACR-TIRADS TR4 (29.24%), EU-TIRADS3 (4.25%), EU-TIRADS4 (22.86%), C-TIRADS4A (16.77%) had higher malignancy rates than the recommended range, but some were comparable to the malignancy rates reported in previous studies (14–16). The difference could be due to the deviation caused by several malignant nodules or sub-centimeter nodules and different observers.

The prerequisite for a guideline to be widely used is that it has good consistency among doctors. In our study, the two doctors used the five guidelines and showed consistent results, indicating that the five guidelines can be used in a standardized manner. Regarding US features, the inter-observer agreement was slightly worse for hyperechoic (ICC, 0.726), echogenicity (ICC, 0.758) and margin (ICC, 0.799) relative to other features. The study by Park et al. (17) concluded that the consistency of echogenicity was poor. In a multi-center study by Persichetti et al. (18) and a single-center study by Giorgio et al. (19), hyperechoic and margin were also US features with poor interobserver agreement. A uniform lexicon of thyroid US features, simplified classification methods, and specialized training to describe thyroid US findings may improve observers’ agreement.

ROC was used to analyze the diagnostic performance of the four guidelines. First, the diagnostic cut-offs of the four guidelines, ACR-TIRADS TR5, Kwak-TIRADS 4C, KTA/KSThR-TIRADS 5, and EU-TIRADS 5 were identified, which was similar to that of Gao, Ali Murat Koc (14, 20). However, Simone indicated that the diagnostic cut-off value of ACR-TIRADS is TR4, while Du showed that the diagnostic cut-off value of Kwak-TIRADS is 4B (21, 22). They also showed that the malignancy rate of the two nodule grades is very high, possibly due to the deviations in the data source. The diagnostic cut-off value of C-TIRADS was 4C. ACR-TIRADS showed the highest specificity compared to the other three guidelines (except C-TIRADS), similar to previous findings (14, 23, 24). The above articles all compared multiple guidelines, including ACR-TIRADS and Kwak-TIRADS. This study shows that Kwak-TIRADS had the highest sensitivity. Hu et al. (25) was similar to this study. C-TIRADS had the highest specificity(82.3%),PPV(69.2%) and accuracy (76.0%). The sensitivity of C-TIRADS (75.7%) was low, the same as Zhu et al., but it was still better than ACR-TIRADS (73.9%) (26). Besides, C-TIRADS had the highest AUC (0.816, all P<0.05). Therefore, C-TIRADS has the highest diagnostic performance under the premise that each diagnostic index had no obvious shortcomings.

This study included 19.1% sub-centimeter nodules. Most guidelines recommend using an active monitoring strategy instead of surgical treatment for low-risk sub-centimeter nodule treatment. However, in China, some patients with sub-centimeter nodules (such as suspicious cervical lymph nodes, other thyroid symptoms, no active follow-up, or hope for more radical treatments)choose surgery. Furthermore, most low-grade sub-centimeter nodules were obtained with malignant nodules when the thyroid lobes were removed. However, these treatment options were controversial. Presently, few studies have reported on various diagnostic properties of sub-centimeter thyroid nodules.

Except for Kwak-TIRADS 4B, C-TIRADS 4B, and 4C, the malignancy rates were no statistical difference between “nodules ≤ 10mm” and “ nodules <10mm” in the other grades (all P>0.05). The AUCs of the five guidelines were not statistically different between the two sizes (all P>0.05). Many studies have shown that the malignancy rate of high-grade small-size nodules is lower than that of large-size nodules. Studies have also shown that the guidelines have better diagnostic efficiency in identifying “nodules <10mm” than “nodules ≤10mm” (12, 14). However, some studies have shown that the incidence of malignant tumors increases with the number of suspicious features, regardless of the size of the nodules (27, 28). Some studies have shown that papillary thyroid microcarcinomas (PTMCs) account for 59.7% of malignant nodules and increase during follow-up (13, 29–31). At present, although the diagnostic ability of the guidelines is controversial for sub-centimeter nodules, it is clear that in our study, the diagnostic ability of the 5 guidelines for “nodules ≤ 10mm” is not inferior to “nodules <10mm”.

This research also had some limitations. First, all patients underwent thyroidectomy, increasing the proportion of malignant nodules, decreasing the number of low-grade nodules, thus increased the number of high-grade nodules. This can cause selection bias, affecting the diagnostic efficacies of the guidelines and reducing the consistencies of diagnoses. Second, clinicians retrospectively analyzed all nodes based on static images only. Static images will affect the evaluation of ultrasonic features, especially the margin of nodules. Real-time dynamic images can evaluate ultrasonic features more accurately. Finally, this was a single-center retrospective study, with guaranteed consistencies of nodule diagnosis results. But, the heterogeneity of the patient population was smaller than that of the multi-center study.

## Conclusions

All five guides showed excellent inter-observer agreement. C-TIRADS was slightly efficient than Kwak-IRADS, KTA/KSThR-TIRADS and ACR-TIRADS, and had greater advantages than EU-TIRADS. The diagnostic abilities of the five guidelines for “nodules ≤ 10mm” were not inferior to that of “nodules> 10mm”. C-TIRADS is simple and easy to implement and can provide effective thyroid tumor risk stratification for thyroid nodule diagnosis, especially in China.

## Data Availability Statement

The original contributions presented in the study are included in the article/**Supplementary Material**. Further inquiries can be directed to the corresponding author.

## Ethics Statement

Ethical review and approval was not required for the study on human participants in accordance with the local legislation and institutional requirements. Written informed consent for participation was not required for this study in accordance with the national legislation and the institutional requirements.

## Author Contributions

AZ and PX collected and classified the thyroid nodules. SG, SC and YL followed up the thyroid nodules. QQ and XH compiled and analyzed the data. QQ and AZ wrote the paper. All authors contributed to the article and approved the submitted version.

## Funding

This study was supported by Key Research and Development Program of Jiangxi Province (20181BBG70031) and Jiangxi Postgraduate Innovation Foundation (YC2021-B043).

## Conflict of Interest

The authors declare that the research was conducted in the absence of any commercial or financial relationships that could be construed as a potential conflict of interest.

## Publisher’s Note

All claims expressed in this article are solely those of the authors and do not necessarily represent those of their affiliated organizations, or those of the publisher, the editors and the reviewers. Any product that may be evaluated in this article, or claim that may be made by its manufacturer, is not guaranteed or endorsed by the publisher.
