# Supplementary material for: Explore the Diagnostic Efficiency of Chinese Thyroid Imaging Reporting and Data Systems by Comparing With the Other Four Systems (ACR TI-RADS, Kwak-TIRADS, KSThR-TIRADS, and EU-TIRADS): A Single-Center Study
Source: Front Endocrinol (Lausanne). 2021 Oct 27;12:763897. doi: 10.3389/fendo.2021.763897 (PMC8578891; doi:10.3389/fendo.2021.763897)
Supplement: Supplementary file 1 [file Table_1.docx]

Supplementary Material

Supplementary Material 1 Clinicopathological and ultrasonic characteristics for thyroid nodules

|  |  |  |  | Total  N(%) |
| --- | --- | --- | --- | --- |
| Multiple nodular glands | No |  |  | 716(81.0%) |
|  | Yes |  |  | 168(19.0%) |
| Hashimoto's thyroiditis | No |  |  | 753(85.2%) |
|  | Yes |  |  | 131(14.8%) |
|  |  | Benign  n(%) | Malignant  n(%) | Total  n(%) |
| Location | Isthmus | 10(1.4%) | 26(6.3%) | 36(3.3%) |
|  | Left lobe | 351(51.5%) | 185(44.7%) | 536(48.9%) |
|  | Right lobe | 321(47.1%) | 203(49.0%) | 524(47.8%) |
| Component | Cystic/almost cystic | 28(4.1%) | 0(0.0%) | 28(2.6%) |
|  | Spongiform | 12(1.8%) | 0(0.0%) | 12(1.1%) |
|  | Mixture of capsule and solid | 218(32.0%) | 6(1.4%) | 224(20.4%) |
|  | Solid/ predominantly solid | 424(62.2%) | 408(98.6%) | 832(75.9%) |
| Echoes | No echo | 28(4.1%) | 0(0.0%) | 28(2.6%) |
|  | Hyperechoic/Isoecho | 277(40.6%) | 20(4.8%) | 297(27.1%) |
|  | Hypoechoic | 344(50.4%) | 323(78.0%) | 667(60.9%) |
|  | Markedly hypoechoic | 33(4.8%) | 71(17.1%) | 104(9.5%) |
| Shape | Wider-than-Tall | 607(89.0%) | 270(65.2%) | 877(80.0%) |
|  | Taller-than-Wide | 75(11.0%) | 144(34.8%) | 219(20.0%) |
| Margin | Well circumscribed | 441(64.7%) | 77(18.6%) | 518(47.2%) |
|  | Microlobulated or irregular | 203(29.8%) | 284(68.6%) | 487(44.4%) |
|  | Ill-defifined | 38(5.6%) | 45(10.9%) | 83(7.6%) |
|  | Extra thyroid extension | 0(0.0%) | 8(1.9%) | 8(0.7%) |
| Hyperechoic | microcalcififications | 137(20.1%) | 199(48.1%) | 336(30.7%) |
|  | Peripheral calcification | 4(0.6%) | 2(0.5%) | 6(0.5%) |
|  | Large calcification | 83(12.2%) | 13(3.1%) | 96(8.8%) |
|  | Comet tail artifacts | 44(6.5%) | 0(0.0%) | 44(4.0%) |
|  | NO | 414(60.7%) | 200(48.3%) | 614(56.0%) |

N=Number of patients

n=Number of nodules

Supplementary Material 2 Unnecessary biopsy rate

| Classification | Nodule size | Benign(n) | Malignant(n) | Total(n) | Unnecessary biopsy Rates(%) |
| --- | --- | --- | --- | --- | --- |
| ACR-TIRADS |  |  |  |  | 50.25 |
| 3 | ≥25mm | 12 | 3 | 15 |  |
| 4 | ≥15mm | 137 | 44 | 181 |  |
| 5 | ≥10mm | 157 | 256 | 413 |  |
| EU-TIRADS |  |  |  |  | 55.99 |
| 3 | ≥20mm | 106 | 4 | 110 |  |
| 4 | ≥15mm | 70 | 20 | 90 |  |
| 5 | ≥10mm | 249 | 310 | 559 |  |
| Kwak-TIRADS |  |  |  |  | 53.09 |
| 4A | ＞10mm | 69 | 6 | 75 |  |
| 4B | ＞10mm | 110 | 21 | 131 |  |
| 4C | ＞10mm | 195 | 278 | 473 |  |
| 5 | ＞10mm | 13 | 37 | 50 |  |
| C-TIRADS |  |  |  |  | 49.02 |
| 4A | ＞15mm | 70 | 12 | 82 |  |
| 4B | ＞10mm | 141 | 95 | 236 |  |
| 4C | ＞10mm | 113 | 224 | 337 |  |
| 5 | ＞10mm | 0 | 6 | 6 |  |
| KTA/KSThR-TIRADS |  |  |  |  | 58.36 |
| 2(spongiform) | ≥20mm | 24 | 0 | 24 |  |
| 3 | ≥15mm | 133 | 6 | 139 |  |
| 4 | ≥10mm | 142 | 50 | 192 |  |
| 5 | ≥10mm | 179 | 285 | 464 |  |
